# Vestibular migraine: an update

**DOI:** 10.1097/WCO.0000000000001257

**Published:** 2024-04-15

**Authors:** Maria D. Villar-Martinez, Peter J. Goadsby

**Affiliations:** a NIHR King's Clinical Research Facility, SLaM Biomedical Research Centre and Wolfson Sensory Pain and Regeneration, Institute of Psychiatry, Psychology and Neuroscience, King's College London, UK; b Department of Neurology, University of California, Los Angeles, California, USA

**Keywords:** migraine phenotypes, postural orthostatic tachycardia syndrome, postural perceptual persistent dizziness, vestibular migraine

## Abstract

**Purpose of review:**

We performed a narrative review of the recent findings in epidemiology, clinical presentation, mechanisms and treatment of vestibular migraine.

**Recent findings:**

Vestibular migraine is an underdiagnosed condition that has a high prevalence among general, headache and neuro-otology clinics. Vestibular migraine has a bimodal presentation probably associated with a hormonal component in women. These patients could have a complex clinical phenotype including concomitant autonomic, inflammatory or connective tissue conditions that have a higher prevalence of psychological symptoms, which may mistakenly lead to a diagnosis of a functional neurological disorder. A high proportion of patients with postural perceptual persistent dizziness have a migraine phenotype. Independently of the clinical presentation and past medical history, patients with the vestibular migraine phenotype can respond to regular migraine preventive treatments, including those targeting the calcitonin gene-related peptide pathways.

**Summary:**

Vestibular migraine is an underdiagnosed migraine phenotype that shares the pathophysiological mechanisms of migraine, with growing interest in recent years. A thorough anamnesis is essential to increase sensitivity in patients with unknown cause of dizziness and migraine treatment should be considered (see supplemental video-abstract).

## INTRODUCTION AND DEFINITION

**FIGURE 1:**
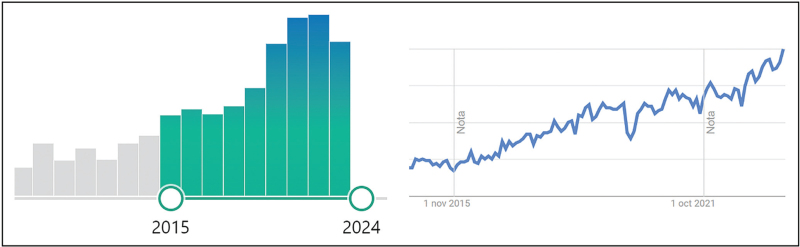
PubMed (left) and Google (right) search trends for ‘vestibular migraine’, accessed 13/12/23. https://trends.google.com/trends/explore?date=all&q=vestibular%20migraine&hl=es.

Vestibular migraine (VM) is probably the second most common cause of dizziness, affecting around 3% of population [1–3]. In recent years, the appearance of new studies aiming to understanding the pathophysiology of VM, alongside increasing interest by the medical and the general population (Fig. 1) may make the disorder even more important [4]. 

**Box 1:**
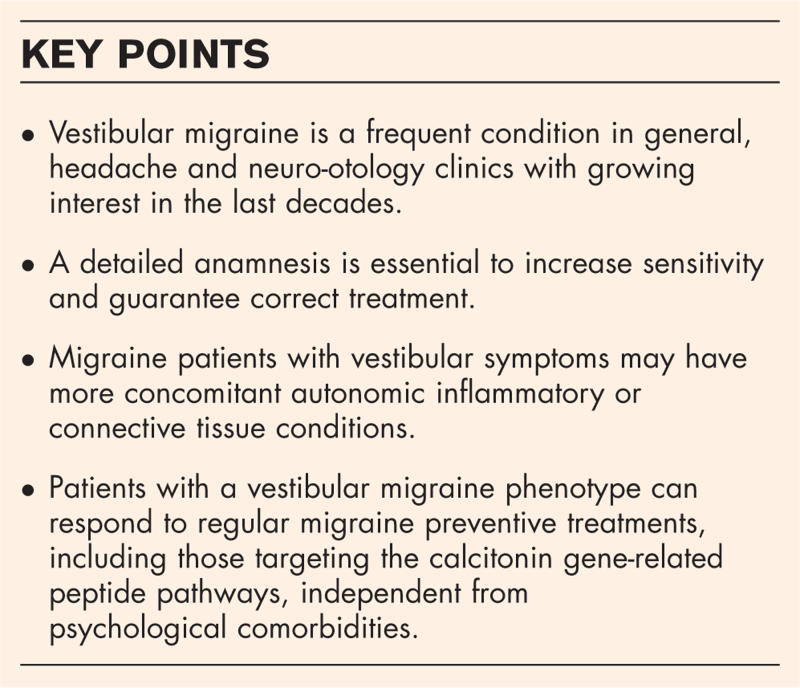
no caption available

Despite this popularity, this condition is far from novelty, as accurately described in antiquity textbooks [5].

The current definition of VM has been available for slightly more than a decade [6]. Phenotyping, collating and defining VM patients has facilitated classification and research, and the term *vestibular migraine* renders a link to its underlying biological mechanisms. Unfortunately, in clinical practice, this etymology may not be ideal, particularly in the nonheadache setting, where the referral reason is dizziness, *vestibular* tests are frequently normal, and there is a high likelihood of receiving a ‘no’ to the question ‘Do you have any headache*?*’. The unconscious association of the word *migraine* with its archetypical manifestations, or considering exclusively the main criteria of the Barany Society and third edition of the International Classification of Headache Disorders (ICHD-3) [6,7] may not be sensitive enough [3,8], ultimately misguiding for the specialist [3]. Instead, the *Notes* section of the classification (Table 1, right) may provide a broader and more accurate clinical picture for milder or atypical phenotypes.

**Table 1:** Main diagnostic criteria (left) and notes (right) of VM (ICHD-3)

A. At least five episodes fulfilling criteria C and D	
*B. A current or past history of Migraine without aura or Migraine with aura*	Code also for the underlying migraine diagnosis
*C. Vestibular symptoms intense enough to either interfere or prevent daily activities, lasting between 5 min to 72 h*	Duration highly variable, lasting for: • Minutes (about 30% of patients) • Hours (30%) • Days (30%) • Seconds only, which tend to happen during head motion, visual stimulation or changes in head position (10%). In these patients, episode duration is defined as the total period during which short attacks recur • There are patients who may take 4 weeks to recover *fully* from an episode
*D. At least half of episodes associated with 1, 2 or 3:* *1. Headache with at least two of the following:* *a) unilateral location* *b) pulsating quality* *c) moderate or severe intensity* *d) aggravation by routine physical activity* *2. Photophobia and phonophobia* *3. Visual aura*	• One symptom is sufficient during a single episode • Different symptoms may occur during different episodes • Associated symptoms may occur before, during or after the vestibular symptoms
*E. Not better accounted for by another ICHD-3 diagnosis or by other vestibular disorder (OVD)*	• History and physical examinations do not suggest OVD • OVD ruled out by appropriate investigations • OVD present as a comorbid condition but episodes can be clearly differentiated • Migraine attacks may be induced by vestibular stimulation • The differential diagnosis should include OVD complicated by superimposed migraine attacks

VM, vestibular migraine.

Additionally, a diagnosis of VM could be confusing for the patient who does not necessarily complain of headache, and received with skepticism, which could potentially hinder compliance to treatment.

Despite the above, naming VM is necessary to avoid increasing the negative predictive value of a diagnosis of psychosomatic, anxiety-related or functional neurological disorder (FND) when the presentation is not typical or upon failure of a first-line migraine preventive treatment. Recognizing a migrainous biology is crucial, given that up to 95% of patients might benefit from migraine treatment, even when headache is not an active symptom [9], and significantly improve quality of life [10].

## EPIDEMIOLOGY – THE SILENT PANDEMIC

The percentage of patients with VM has been described to be between 7% and 16% [1,4,11–13] in neurology and neuro-otology clinics. In the general population in the United States, around 12% of the respondents disclosed dizziness or balance problems, among which 23.4% met the authors’ definition criteria for VM, which accounts for a prevalence of approximately 3% of adults [13]. Indeed, up to 60% of participants with chronic migraine can fulfill VM criteria, and this percentage can increase up to 73% in those with migraine with aura [14^▪^].

Recurrent episodes of vertigo of unknown cause should only be attributed to migraine after a thorough headache anamnesis [15]. Therefore, VM should be included in the differential diagnosis of every patient with unexplained dizziness. During the clinical interview in headache clinics, around 20% of patients spontaneously commented vestibular symptoms accompanying the headache [16]. However, when enquired specifically using a questionnaire, this percentage can double [2,17,18], and even increase up to 75% when asked directly by a clinician. Among migraine patients, 33% also described isolated vertigo episodes outside the headache attack [12].

## CLINICAL PRESENTATION AND MECHANISMS – THE CHAMELEON

Perhaps oversimplified, the mechanisms behind VM seem to be related to vestibulo-trigeminal interactions [19] that may facilitate central sensitization of higher order brain structures [20].

The wide array of manifestations reported in VM [7] has prompted a denotation of ‘chameleonic’ [21]. The inherent difficulty of describing subjective and complex dizziness, vertigo or disequilibrium sensations can make arduous for the patients to explain themselves, who may often select eccentric comparatives, far from the expected ‘room spinning’, such as ‘stepping into a hole’ or ‘being inside a barrel’. Bizarre symptoms without a clear pathophysiological correlation, can be perplexing for the clinician, and despairing for the patients, who may find that their ailments are being dismissed like Cassandra predicting the fall of Troy. Clinical biases may be bigger in the context of psychiatric comorbidities, personality disorders or triggered by stress, with a higher likelihood a receiving potentially irreversible label of FND. Historical examples such as the complicated figure of Martin Luther are illustrative, who mentioned ‘nobody believes me’ in his chronicles, when describing complex vertiginous symptoms triggered by stressful situations [22].

In the absence of reliable VM biomarkers [23,24], the reasonable way forward for an accurate diagnosis is a thorough anamnesis.

### Age

Temporal patterns may produce different phenotypes [20]. The modal distribution of the age of onset of vestibular symptoms varies between biological sexes, peri-menopausal in women, and in the third decade in men [25]. Indeed, menopause was one of the variables distinguishing VM from other migraine phenotypes [26^▪^]. Peri-menopausal change or disappearance of headache has been described in the literature [27], which may be substituted by vestibular symptoms [20,25,28]. Hormonal influence in women is not surprising given the extensive expression of receptors in key areas such as the hypothalamus, brainstem, cerebellum or trigeminal pathways [29]. The low recruitment of postmenopausal women in trials [30] hinders a proper characterization of this natural evolution.

### Frequency

Vestibular symptoms, similar to other associated symptoms to migraine [31^▪▪^], can occur any time before, during or after an attack, and also interictally [25]. Thalamic activation in patients with VM was significantly correlated with attack frequency [32]. In a survey in patients with migraine, they reported a mean duration of 31.9 min and frequency of around 3 monthly episodes that was higher in patients with chronic migraine, headache frequency or severity and longer headache duration. Dizziness or vertigo also correlated with higher levels of depression, anxiety, and disability [2]. Functional neuroimaging has shown differences in the visual, cognitive and pain-perception areas in patients with VM, which, similarly to the general population of migraine patients [33], may be a consequence of repeated attacks [34]. Chronification may cause morphological changes in brainstem areas such as the trigeminal and vestibular nucleus, in which glutamatergic neurons and calcitonin gene-related peptide (CGRP) have shown to play a key role [35,36^▪^]. These alterations would explain the good response, also in patients with aura, to flunarizine, amitriptyline, propranolol [37] or the nutraceutical, magnesium [38].

**FIGURE 2:**
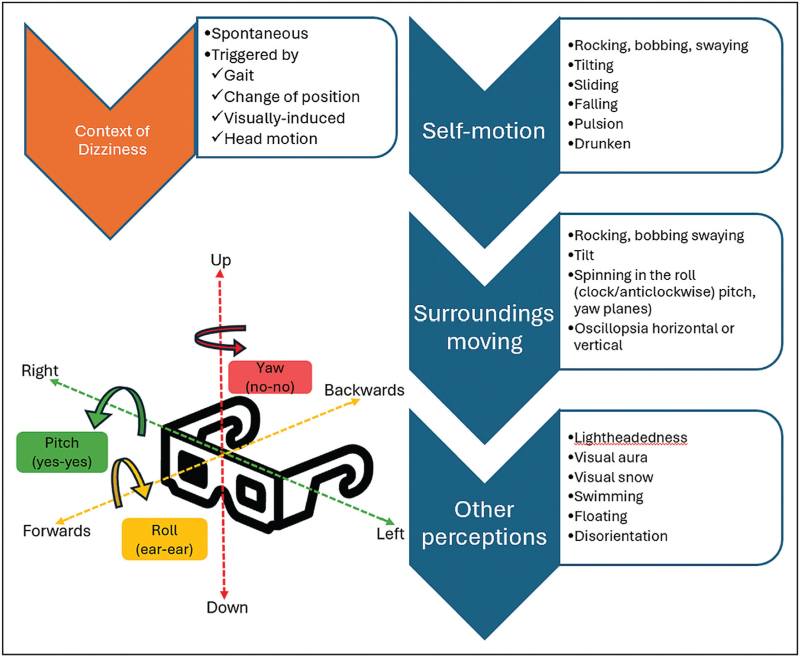
. Clinical presentation of dizziness in patients with migraine. Columns on the right: frequent clinical perceptions reported by migraine patients. Top-left: different contexts that surround the episode of dizziness. Bottom left: different planes and directions of self-movement perceived by the patient.

Patients with VM may present with a constellation of sensations (Fig. 2) [39–44], likely representing alterations in the vestibulo-thalamic-cerebellar networks [45], and the characterization of these is essential to understand the processes behind its pathophysiology [46].

Describing vertiginous symptoms is fundamental in the main differential diagnosis and treatment of vestibular disorders, including benign positional paroxysmal vertigo (BPPV), Meniere's disease (MD), vestibular neuritis, motion sickness, posterior circulation ischemia, vestibular paroxysmia, mal de debarquement, motorist disorientation syndrome, or episodic ataxia [47,48]. Aids such as the SO STONED questionnaire [49] could help differentiating the symptoms in the busy clinic setting.

### Headache and migraine features

VM patients frequently describe not so much a ‘headache’, but a ‘pressure’, uni- or bilaterally in different areas [25] including the skull, trigeminal branches V1–V3, occipital region, or the neck. The latter prompted a recent multidisciplinary expert consensus from the Barany Society that agreed that migraine was the commonest cause for the combination of neck pain and vestibular symptoms [50^▪▪^]. This is clinically unsurprising, given that neck stiffness is the second most common premonitory-like symptom reported and triggered in migraine [51]. Interestingly, when a migraine attack is triggered with nitroglycerin, vestibular symptoms were elicited in 75% of patients who reported vestibular symptoms during a spontaneous migraine attack, and reproducible in 40% [52]. Identifiable attack triggers include, similar to other migraine phenotypes, stress or weather changes [2].

In patients with aura, the frequency of vertigo symptoms is double [2]. Associated features such as photophobia may not be evident in the clinical interview, but the patient may describe, for example, that the fluorescent light of the clinic room is ‘awful’. Structural neuroimaging has demonstrated volume abnormalities in nociceptive and multisensory vestibular brain areas in patients with VM, which were not present in regular migraine patients [53]. VM patients, as well as migraine patients, can be more sensitive to certain stimuli. Visual symptoms simulating motion sickness [41] may trigger classic associated symptoms to migraine, such as nausea and skin tenderness in population with migraine [54], which are of similar intensity and duration of those with vestibular migraine [55]. Increased sensitivity may manifest beyond the central nervous system, such as certain abnormal bodily reactions to drugs and other agents [56].

### Comorbidities

Patients who complain of several ailments can be challenging in the short slots of a clinical environment, however, when patients are allowed to speak freely, symptoms can present a pattern. A study of consecutive patients attending a headache clinic found almost 60% of patients with regular migraine had vestibulo-cochlear symptoms [25], some of which can be triggered with well known migraine triggers such as nitroglycerin [57^▪^]. Migraineurs have a higher probability of presenting other vertiginous conditions such as benign positional paroxysmal vertigo (BPPV), motion sickness or MD, among others [1,58]. Besides, migraine patients have a higher risk to develop ear problems such as tinnitus or hearing loss [59,60^▪^], which may lead to misdiagnosis, even in specialized clinics [28]. The clinical picture of an attack of MD can be so similar to that of VM, with overlaying symptoms including cochlear disturbances, tachycardia, vomiting or even photophobia, which may render this indistinguishable for the expert physician [61]. Again, CGRP may be related to the manifestations of these symptoms, as a significant amelioration has been reported with erenumab in overlapping phenotypes [62].

**FIGURE 3:**
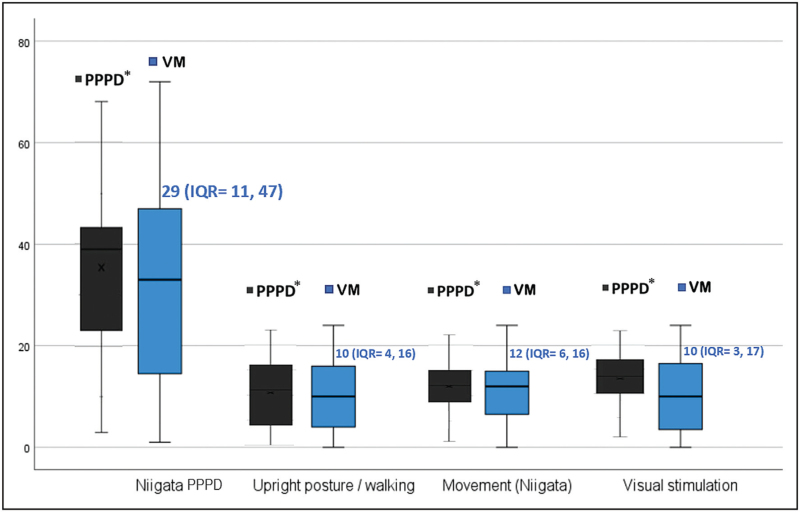
Results comparison using the Niigata questionnaire in Persistent Postural Perceptual Dizziness and patients with migraine. IQR, interquartile range [71].

In 1937, Papez described that emotions arise not only from psychic activity, but also from activation of the hypothalamic region [63]. Patients diagnosed with persistent postural perceptual dizziness (PPPD) parallel migraine in multimodal hypersensitivity, altered functional connectivity between thalamic, visual, vestibular, limbic and cerebellar regions, which may generate excessively dependent on visual and postural stimuli [64,65,66^▪^]. When vestibular migraine chronifies, one may misinterpret daily or persistent symptoms for those of PPPD, considered an FND [67,68]. A recent study found that more than half of the patients with PPPD, could meet criteria for migraine, and up to 17% for definite VM [69]. Likewise, almost 40% of migraineurs fulfill PPPD criteria [70]. The graphical comparison of the results of Niigata's questionnaire, a validated tool used to diagnosed PPPD, was almost identical between PPPD and VM (Fig. 3) [71].

Other systemic comorbidities linked to VM may shed light on its biology. In 1975 ‘Chronic neurovisceral dysfunction’ was coined to describe an conglomerate of features, among which vertigo or dizziness were usual, including asthma, bladder problems, sleep disturbances, rhinitis, tachycardia, gastrointestinal disorders and memory problems of potential central origin involving substances such as serotonin, histamine or mast cells [72].

Motion sickness in migraineurs responds to treatment with serotonin agonists such as rizatriptan [73]. The metabolism of serotonin's precursor tryptophan seems to be altered in migraine [74], and is abundant in foods attributed to ‘trigger’ an attack, which may rather represent storing during the premonitory phase [75,76]. It has been speculated that neuropeptide Y could be a therapeutic target during this phase, as it is localized in areas such as the hypothalamus, basal ganglia and limbic system, and involved in appetite control [77]. Mast cells and histamine may also be involved in central pathways and external manifestations of cranial autonomic symptoms [78,79].

The presence of autonomic, inflammatory conditions and hypermobility conditions was higher in a recent study comparing patients with VM with chronic migraine patients. Specifically, the prevalence of Postural Orthostatic Tachycardia Syndrome quintupled that of the general population [80].

Other conditions summarized in Table 2, [81,82] including inflammatory [79,83,84] connective tissue [85–87] and other disorders [88–90] usually linked to migraine also in pediatric populations [91,92], manifest higher vulnerability to develop further psychological symptoms [93].

**Table 2:** Examples of pain, inflammatory, autonomic and connective tissue comorbidities that could be present in patients with VM and psychological comorbidities

A	Autonomic problems: POTS, orthostatic hypotension, unexplained syncope
M	Migraine associated symptoms: photo, phono, osmophobia, allodynia
I	Inflammatory: asthma, rhinitis, thyroid disease, allergies
G	Gastro intestinal: GERD, gastritis, hiatus hernia, IBS, intestinal inflammatory disease
U	Uterine problems: endometriosis, polyps, myomas, hysterectomy
I	Incontinence: hyperactive bladder, frequent cystitis
T	Tissue: hypermobility, EDS, RA, SLE, scleroderma, PMO/DMM, Sjogren
A	Algias: fibromyalgia, chronic back/pelvic pain, joint pain, chest pain
S	Skin problems: urticaria, psoriasis, eczema, rashes, atopy, hives

DMM, distal musculoskeletal manifestations; EDS, Ehler-Danlos syndrome; GERD, gastro-esophageal reflux disease; IBS, irritable bowel syndrome; PMR, polymyalgia rheumatica; POTS, postural orthostatic tachycardia syndrome; RA, rheumatoid arthritis; SLE, systemic lupus erythematosus; VM, vestibular migraine.

Inflammatory pathways in migraine are not clear, and may differ between males and females, as described in preclinical studies, in which stress and anxiety, paradoxically, did not influence female behavior [94,95].

Patients with MD overlapping phenotype could have a more inflammatory background [96]. A relationship between dysautonomia, hypermobility syndromes, or the currently poorly classified and understood mast-cell activation syndrome [97^▪^] could conceivably justify why a sub-population of migraineurs with a dizzy phenotype have, among their daily medication, an antihistamine such as loratadine or cetirizine.

## INVESTIGATIONS

Other vestibular disorders should be ruled out by appropriate investigations, as patients may present two different independent conditions with episodes easily differentiated [6]. Audio-vestibular tests and neuroimaging should be requested in every patient with new-onset vestibular symptoms [98]. Abnormal electronystagmography can be found in up to 80% of patients with migraine [72,99], with electroencephalographic alterations that seem to be ipsilateral [72]. Lower percentages have been reported in younger populations with less proportion of females [100].

Interictally, patients with VM have abnormalities in vestibular tests, also in a lower proportion without vertigo complaints [101]. Stimulation of supraorbital branches of the trigeminal nerve elicits changes in nystagmography not present when stimulating other extra-cranial nerves [102].

Brain MRI and other sequences depending on the patient's presentation should be requested in all patients with new-onset vestibular symptoms, such as inner ear or brainstem specific sequences [103^▪^].

## TREATMENT

Completed studies using standard methods have shown that migraine treatments can also be effective for VM. Table 3 includes a variety of pharmacological options recommended in international guidelines [104,105], which have shown to be effective also for vestibular migraine, although, as discussed in recent systematic reviews [106–108], these studies have low quality and there is enormous methodology heterogeneity. It is however remarkable that, independent of the fulfillment of the current Barany criteria, when grouped by symptoms, i.e. vertigo, disequilibrium or both, response to a migraine preventive did not significantly differ in any of the groups [9], or that reduction of vertiginous symptoms is independent from presenting psychiatric comorbidities, as an amelioration in vertigo frequency or severity is not necessarily correlated with the improvement in anxiety or depression when comparing antidepressants with other preventives [109]. Patients with previous failure in several preventive treatments may also benefit from longer treatment with next-line prevention, such as medication targeting the CGRP pathway [110,111]. Populations with high proportion of aura had a good response to symptomatic treatment with flunarizine [112], and brainstem aura seems to respond to the similarly-structured drug, cinnarizine [113]. Lifestyle or dietary modifications should be discussed when appropriate.

**Table 3:** Migraine pharmacological treatments, effective in vestibular migraine

Reference	Year	Study type	Follow-up time	Preventive	Dose	*N*	Vertigo active	Vertigo control	Headache active	Headache control
Reploeg	2002	Observational, sequential	>1 month	Nortriptyline + diet	10–50 mg	31	31 Resolution or 75% reduction in frequency	13 Resolution with diet	NA	NA
			>1 month	Nortriptyline + Atenolol	25–50 mg	19	21 Reduction or 75% reduction in Frequency			
			>1 month	Nortriptyline + Atenolol + Calcium-channel blocker + Neurology review	UNK	18				
Bisdorff	2004	Observational, open-Label	3–4 months	Lamotrigine	100 mg	19	Frequency reduced from 18 to 5	NA	Frequency reduced from 9 to 4	NA
Gode	2010	Randomised, open-label	6 months	Topiramate	50 vs. 100 mg	26	Reduction in vertigo frequency and severity (*P* < 0.05)	Reduction in headache frequency and severity (*P* < 0.05)		
Mikulec	2012	Retrospective, sequential	UNK	Nortriptyline vs. diet modification	25–75 mg	24	Reduction in dizziness in 46% (*P* < 0.05)	Reduction in 14% of participants (US)		Reduction in 14% of participants (US)
			UNK	Topiramate vs. diet modification	50–100 mg	22	Reduction in dizziness in 25% (*P* = 0.442)	Reduction in 14% of participants (US)	Reduction in headache in 25% (*P* = 0.442)	Reduction in 14% of participants (US)
Lepcha	2013	Open-label	3 months	Flunarizine + (betahistine + paracetamol during attacks)	10 (F), 16 (B), 1000 (P) mg	25	Reduced frequency and severity of vertigo (*P* < 0.05)	NA	NS	NA
Taghdiri	2014	Retrospective	3 months	Cinnarizine	37.5–75 mg	24	Reduction in attack frequency *(P* < 0.05)	NA	Reduction in Frequency, duration and severity *(P* < 0.05)	NA
Van Ombergen	2014	Retrospective	UNK	Flunarizine	10 mg	30	Improvement in VM symptoms in 68% (*P* < 0.001)	NA	NA	NA
			UNK	Propranolol	80 mg	31	Improvement in VM symptoms in 73% (*P* < 0.001)	NA	NA	NA
Salviz	2015	Randomised, open-label	4 months	Propranolol	40–160 mg	26	Reduction in DHE, attack frequency and VAS (NS between groups)	Reduction in DHE, attack frequency and VSS (NS between groups)	NA	NA
		Randomised, open-label	4 months	Venlafaxine	37.5–150 mg	26	Reduction in DHE, attack frequency and VAS (NS between groups)	Reduction in DHE, attack frequency and VSS (NS between groups)	NA	NA
Teggi	2015	Observational, Open-Label	6 months: three times 1 month active drug intercalated with 1 month without	Cinnarizine & Dimenhydrinate vs. Lifestyle measures	20 mg (C), 40 mg (D)	22	Reduction in vertigo frequency (*P* < 0.001)	Reduction in vertigo frequency (*P* < 0.05)	Reduction in vertigo frequency (*P* < 0.001)	Reduction in vertigo frequency (*P* = 0.06)
Salmito	2016	Retrospective	3 months	Amitriptyline	20–50 mg	15	Reduction in VAS vestibular symptoms (*P* < 0.001), similar between groups	NA	Reduction in VAS vestibular symptoms (*P* < 0.001), similar between groups	NA
				Flunarizine	10 mg	11				
				Propranolol	40–80 mg	7				
				Topiramate	100–200 mg	8				
Yuan	2016	Randomised	3 months	Flunarizine and betahistine	12 mg (B) ±10 mg (F)	23	Reduction in frequency, duration and severity (*P* < 0.05)	Reduction in frequency, duration and severity (*P* < 0.05)	NA	NA
Liu	2017	Single-blinded	3 months	Venlafaxine	37.5 mg	23	Reduced DHI (*P* < 0.05) attack frequency and VSS (*P* < 0.001)	NA	NA	NA
			3 months	Flunarizine	10 mg	22	Reduced DHI-f,p,t and VSS (*P* < 0.05) but not frequency (*P* = 0.057)	NA	NA	NA
			3 months	Valproic Acid	1000 mg	20	Reduced DHI-f,p,t (*P* < 0.05) and Frequency (*P* < 0.001), but no VSS (*P* = 0.27)	NA	NA	NA
Bayer	2019	Randomized, placebo-controlled	6 months	Metoprolol	95 mg	60	Nonsignificant reduction in additional monthly rate of attack incidence	Reduction in additional monthly rate of attack incidence	Headache frequency not obtained at baseline	Headache frequency not obtained at baseline
Celik	2020	Prospective, open-label	6–32 months	Propranolol	40–60 mg	38	Reduction of DHI and VADL (*P* < 0.001)	NA	NA	NA
Dominguez-Duran	2020	Randomized per algorithm, open-Label	5 weeks	Acetazolamide	250 mg	5	Reduction in frequency and VAS vestibular symptoms (*P* < 0.001), similar between groups	NA	Reduction in headache symptoms per VAS (*P* < 0.001), similar between groups	NA
				Amitriptyline	10 mg	16				
				Flunarizine	5 mg	1				
				Propranolol	10 mg	4				
				Topiramate	25 mg	5				
Gorur	2020	Open-label	3 months	Botox + (A, P, F)	155 IU	10, 11, 9	Reduced vertigo frequency and DHI (*P* < 0.001)	NA	Reduced MIDAS (*P* < 0.001)	NA
			3 months	Propranolol	≤80 mg	9	Reduced vertigo frequency and DHI (*P* < 0.001)	NA	Reduced MIDAS (*P* < 0.001)	NA
			3 months	Amitriptyline	25–75 mg	10	Reduced vertigo frequency and DHI (*P* < 0.001)	NA	Reduced MIDAS (*P* < 0.001)	NA
			3 months	Flunarizine	10 mg	11	Reduced vertigo frequency and DHI *(P* < 0.001)	NA	Reduced MIDAS (*P* < 0.001)	NA
Hoskin	2022	Retrospective	NS	Erenumab	NS	11	Improvement in 21/25, moderate-significant in 15/25 (NS)	NA	Improvement in 21/25, moderate-significant in 15/25 (NS)	NA
				Fremanezumab		9				
				Galcanezumab		6				
				Ubrogepant		2				
Lovato	2023	Observational, open-Label	Mean 26 weeks	Erenumab vs. previous preventives	140 mg	23	Reduction in DHI, (*P* *<* 0.001) and VNG abnormalities (*P* = 0.002)	NA	Reduction in mean migraine days (*P* = 0.001)	NA
			Mean 28 weeks	Propranolol	40–80 mg	12	Reduction in DHI, (*P* *=* 0.2)	NA	Reduction in mean migraine days (*P* = 0.2)	NA
				Flunarizine	5 mg	7				
				Valproic acid	500–800 mg	4				
Russo	2023	Prospective, open-label	12 months	Erenumab	140 mg	7	Reduction in mean monthly days with dizziness (*P* < 0.001)	NA	Reduction in migraine monthly frequency (*P* < 0.001)	NA
				Fremanezumab	225 mg	25				
				Galcanezumab	240 initial, then 120 mg	18				

DHI, Dizziness Handicap Inventory; E emotional; F functional; NA not applicable; NS, not significant; P physical; VAS, visual analogue scale; VM, vestibular migraine.

When evaluating the need of symptomatic and acute treatment, associated symptom should always be characterized, and treated when possible [31^▪▪^]. Despite a large number of studies assessing the role of preventive treatment in VM, systematic reviews on symptomatic treatment for vestibular migraine have been disappointingly negative and thus not promising from a practical perspective [114]. The low power in these studies may be attributed to patients not fulfilling strict criteria [115].

**FIGURE 4:**
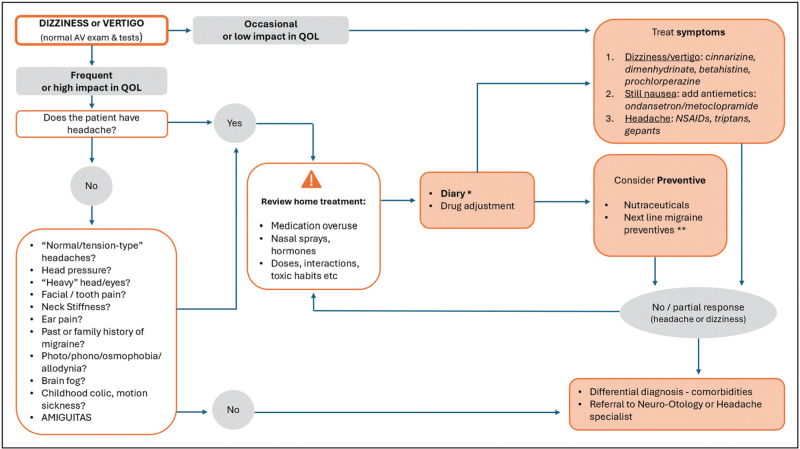
Work-up for the management of the dizzy patient to increase sensitivity of vestibular migraine. ∗ Diary for headache, dizziness and menstruation if applicable. ∗∗ Preventive treatment to be started in consultation with the patient: adverse effects, comorbidities and biopsychosocial circumstances. Initial dose below the minimum recommended therapeutic doses in patients with hypersensitivities/allergies. AMIGUITAS, autonomic, migraine associated symptoms, inflammatory, gastro-intestinal, uterine, incontinence, tissue, Algias, skin problems; AV, audiovestibular; NSAIDs, nonsteroidal anti-inflammatory drugs; QOL, quality of life.

Figure 4 shows an algorithm for the diagnosis and management of VM.

## CONCLUSION AND FUTURE

The current understanding of vestibular migraine is likely to grow substantially in the near future. Patients with migraine and vestibular symptoms may present autonomic, inflammatory or pain concurrent comorbidities, and respond to migraine preventive treatment independent of psychological symptoms. Formulating a thorough anamnesis is fundamental to prevent an unnecessary label of a functional diagnosis. Further research focusing on understanding the different individual phenomena of this migraine phenotype is needed.

## Acknowledgements

*We would like to express our gratitude to David Cheung for his bibliographic assistance to this manuscript and Kostis Christoforou for his audiovisual support.*

### Financial support and sponsorship

*The authors did not receive any external funding for this manuscript.*

### Conflicts of interest

*PJG reports, over the last 36 months, grants from Celgene and Kallyope, and personal fees from Aeon Biopharma, Abbvie, Amgen, eNeura, CoolTech LLC, Dr Reddys’, Eli-Lilly and Company, Epalex, Linpharma, Lundbeck, Man&Science, Novartis, Pfizer, Sanofi, Satsuma, Shiratronics, and Teva Pharmaceuticals, and personal fees for advice through Gerson Lehrman Group, Guidepoint, SAI Med Partners, Vector Metric, and fees for educational materials from CME Outfitters, and publishing royalties or fees from Massachusetts Medical Society, Oxford University Press, UptoDate and Wolters Kluwer, and a patent magnetic stimulation for headache (No. WO2016090333 A1) assigned to eNeura without fee. M.D.V.M. has no conflicts of interest.*

## REFERENCES AND RECOMMENDED READING

Papers of particular interest, published within the annual period of review, have been highlighted as:

▪ of special interest
▪▪ of outstanding interest
